# Recent Advances in Immune Cell Therapy for Glioblastoma

**DOI:** 10.3389/fimmu.2020.544563

**Published:** 2020-10-21

**Authors:** Xianhui Kang, Yiyang Zheng, Wandong Hong, Xixi Chen, Huiting Li, Baojun Huang, Zhenyang Huang, Hongli Tang, Wujun Geng

**Affiliations:** ^1^Department of Anesthesiology, The First Affiliated Hospital of Wenzhou Medical University, Wenzhou, China; ^2^Department of Anesthesiology, The First Affiliated Hospital, College of Medicine, Zhejiang University, Hangzhou, China; ^3^First School of Clinical Medicine, Wenzhou Medical University, Wenzhou, China; ^4^Department of Gastroenterology and Hepatology, The First Affiliated Hospital of Wenzhou Medical University, Wenzhou, China

**Keywords:** glioblastoma, immunotherapy, immune cell, advances, mechanism

## Abstract

Glioblastoma (GBM) is the most malignant form of astrocytoma with short survival and a high recurrence rate and remains a global problem. Currently, surgery, chemotherapy, radiotherapy, and other comprehensive treatments are the main treatment modalities, but patients still have a poor prognosis mainly due to the infiltrative growth of GBM and the protective effect of the blood–brain barrier on tumor cells. Therefore, immunotherapy is expected to be a good option for GBM. In the immune system, different cells play varying roles in the treatment of GBM, so understanding the roles played by various immune cells in treating GBM and considering how to combine these effects to maximize the efficacy of these cells is important for the selection of comprehensive and optimal treatment plans and improving GBM prognosis. Therefore, this study reviews the latest research progress on the role of various types of immune cells in the treatment of GBM.

## Introduction

### Glioblastoma

Glioblastoma (GBM) is a rare tumor that is one of the most fatal and difficult-to-treat malignancies. Currently, the primary treatment for GBM is still based on surgery, and patients usually have a poor prognosis and poor quality of life (1). The tumor is subcortical, and most grow throughout the supratentorial cerebral hemispheres. It exhibits infiltrative growth, often invades several lobes and deep structures, and has been shown to affect the contralateral cerebral hemisphere through the corpus callosum with the frontal lobe being the most common site of occurrence (2). GBM grows rapidly with 70% to 80% of patients dying of GBM within 3 to 6 months after diagnosis and a 1-year survival rate of only 10%.

### Immune Cell Therapy

There is a large body of literature demonstrating that immunotherapy is important for the treatment of GBM. Chimeric antigen receptor (CAR) T cell therapy can directly and accurately identify, localize to, and kill cancer cells. Natural killer (NK) cells control GBM expansion and inhibit tumor progression; dendritic cells (DCs) play a role in GBM immune recognition, and other immune cells play an adjuvant role in radiotherapy and chemotherapy treatments for GBM.

The main feature of GBM metastasis is extensive local invasion, which is different from the rarer event of systemic metastasis. Therefore, cancer immunotherapy at this stage focuses more on the use of immune cells to inhibit the metastasis of GBM.

#### Advantages of Immune Cell Therapy

Some immune cells with recognition functions can distinguish themselves from nonself cells (which present nonself antigens), providing a great opportunity for the use of immune cells to specifically recognize and kill cancer cells.

Although the metastatic spread of GBM is extremely rare, GBMs can grow in areas of the brain that are hard to access surgically. Compared with the gross removal of tissue *via* surgical resection, the specific recognition and killing ability of immune cells is more likely to remove only the cancer cells, which has great advantages over less specific treatment modalities.

#### Disadvantages of Immunotherapy

GBM is highly susceptible to recurrence, and most recurrent tumors have been subjected to genotoxic stress from radiotherapy and/or chemotherapy and are, thus, more immunogenic than untreated tumors (3). However, because recurrent gliomas often engage in antigen escape after immunotherapy, it is difficult to perform immunotherapy on these tumors.

## Changes in Associated Immune System After GBM Development

**Figure 1:**
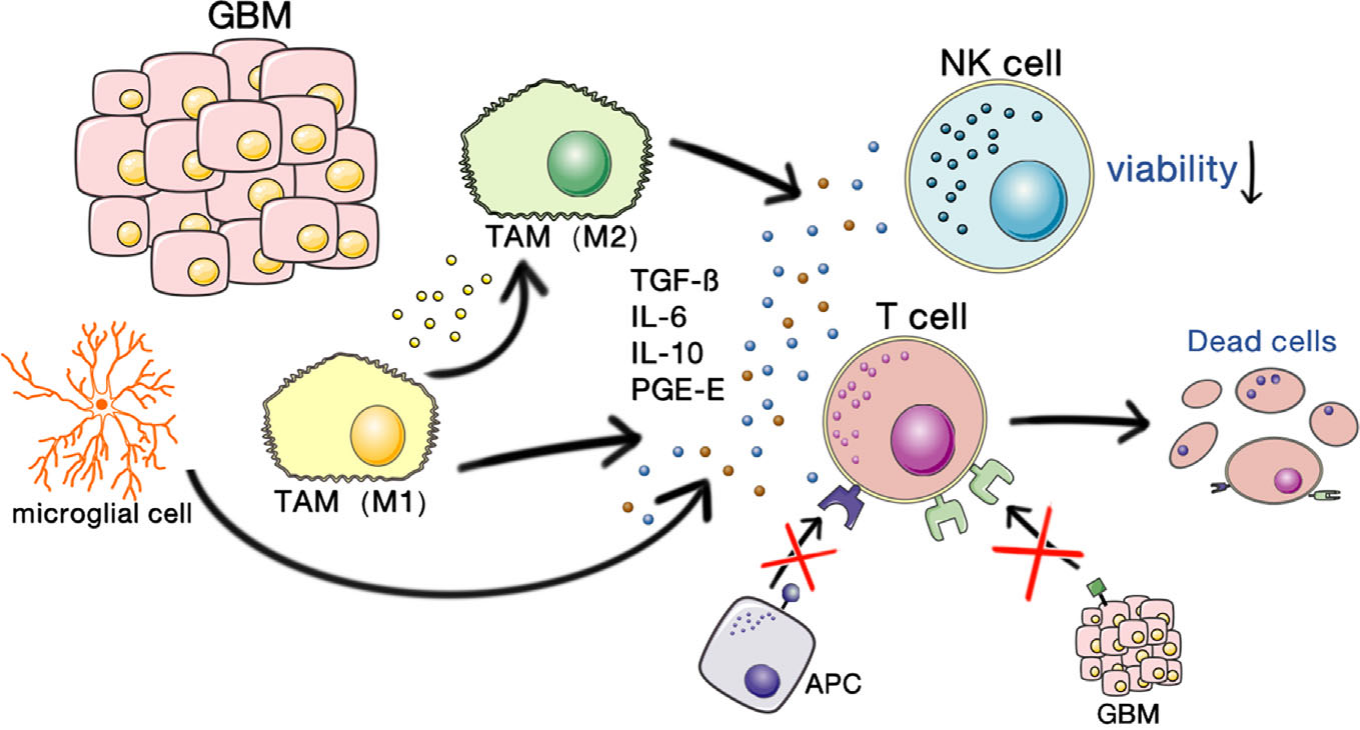
Immunosuppressive microenvironment of GBM. GBM-associated macrophages and microglia secrete inhibitory cytokines, which decrease NK cell activity and T cell–mediated apoptosis and inhibit the binding and killing effects of T cells on antigen-presenting cells and GBM cells. This allows the tumor to escape the immune-killing effects of NK cells and T cells.

Because GBM occurs in the brain, the immunosuppression of GBM involves both the tumor itself and the unique immune characteristics of the brain. The interactions of glioma stem cells (GSCs) and the tumor microenvironment play vital roles in promoting the malignant growth of GBMs. A schematic illustrating the immunosuppressive microenvironment in GBM is shown in **Figure 1**.

### Brain Autoimmune Properties

The blood–brain barrier (BBB) is an important line of defense for brain immunity. The BBB is an astrocyte-supported network of tight junctions on the endothelium that prevents the diffusion of hydrophilic macromolecules into the CNS while allowing the entry of small hydrophobic molecules and the active transport of glucose and nutrients (4).

### The Immune Microenvironment of GBM

#### Glioma Vasculature

The vasculature within gliomas shows upregulated protein expression of the macromolecules periostin and tenascin C (TNC), which can prevent T cells from moving into glioma-associated vessels and prevent their migration into the brain parenchyma (5).

#### Upregulation of Immunosuppressive Molecules (Immune Checkpoints)

Immune checkpoints are small molecules present on the cell surface of T lymphocytes that maintain immune homeostasis. Some immune checkpoint genes, such as CTLA-4, PD-1, LAG3, TIM, and BTLA, mediate inhibitory signals, thereby inhibiting T cell activity (6). The expression of CTLA-4 and PD-1 in GBM often rises immensely, which suppresses immunity (3).

#### Soluble Factors (e.g., Cytokines and Growth Factors)

The soluble factors TGFβ, IL-10, and prostaglandin 50 were the earliest immunosuppressive mediators identified in GBM patients. TGF-ßTME and IL-10 cause microglia to lose their MHC expression (5).

#### Tumor-Associated Immunosuppressive Cells

GBM is characterized by the infiltration of microglia and peripherally recruited macrophages, whereas lymphocytic infiltration is usually low (7). Tumor-associated macrophages (TAMs) secrete inhibitory cytokines, such as interleukin-6 (IL-6), IL-10, transforming growth factor β (TGF-β), and prostaglandin-E, which inhibit NK cell activity and the activation and proliferation of T cells and induce T cell apoptosis, thereby downregulating the expression of MHC and changing TAMs to the M2 phenotype, resulting in immunosuppression (3).

## Immune Cell Therapy for GBM

### Role of NK Cells in the Treatment of GBM

NK cells are the first natural line of defense against infection and antitumor immunity, and their surface inhibitory receptors recognize MHC class I molecules on the surface of normal somatic cells. When somatic cells are mutated (e.g., GBM), MHC class I expression on their surface is lost, and NK cells initiate a killing effect.

NK cells are persistent in targeting tumor cells and are difficult to escape, and current studies focus on mimicking NK cell activity to replicate their attacking and immune-killing effects (8).

**Figure 2:**
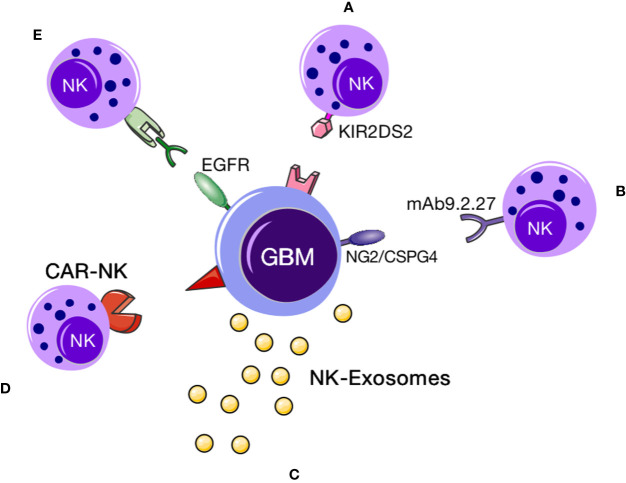
NK cell immunotherapy. NK cell–based immunotherapy for GBM. This figure demonstrates that a variety of NK cell therapies for GBM. **(A)** KIR2DS2 immunotype NK cells could target and destroy GBM cells (10); **(B)** exosomes secreted by NK cells could specifically localize to GBM cells, upon which the cytokines within the exosomes could induce apoptotic signaling in tumor cells to promote cell death; **(C)** CAR-NK immunotherapy comprising NK cells expressing a GBM-specific CAR can target the tumor; **(D)** specific proteins (such as CD16) can bind NK cells and EGFR on the surface of GBM cells to facilitate NK cell activity. **(E)** using specific proteins (such as CD16) to bind NK cells and then specifically bind to EGFR on the surface of glioblastoma cells to exert a cytotoxic effect.

The applications of NK cell therapy for GBM can be summarized as follows: 1. direct use of NK cells to kill GBM cells, 2. combined immune cell therapy regimens comprising NK cells and immune checkpoint inhibitors or drugs targeting immune-related genes or specific antibodies targeting proteins that protect against immunosuppression of NK cells, and 3. chimeric antigen-modified NK (CAR-NK) cell therapy (9). Images of NK cell–based immunotherapies are shown in **Figure 2**.

NK cells prevent systemic metastasis of GBM. If NK cells are transplanted into a GBM model, GBM death can be directly induced (11), but the difficulty of this method lies in the uncertainty of the transplantation process of the NK cells. It has been suggested that GBM occurs due to cytomegalovirus infection interfering with the immune response of NK cells (12).

Yvon et al. (13) propose an immunotherapy approach for GBM using NK cells derived from cord blood, but this method is similarly limited by immunosuppressive cytokines in the tumor microenvironment. In addition, there have been some studies proposing combination immunotherapies related to NK cells. For example, by using the addition of the immune checkpoint inhibitor PD-1 combined antibody, one group found that decreased PD-1 activity could promote the massive infiltration of NK cells and T cells as well as inhibit tumor progression (14). However, none of these combination immunotherapies have been studied with regard to the specific mechanism of action of other cells beyond NK cells to help kill GBM.

Systemic metastasis of GBM is rare thanks to the innate immunity of NK cells, and studying the combination of radiotherapy, immune cells, and immune checkpoint inhibitors is beneficial to improve the treatment of GBM. Based on the above studies, although a large amount of experimental data show that the above methods can increase immune cell infiltration, there are still many problems in the clinical application of this regimen. Therefore, the use of NK cells in the brain to kill GBM still has a series of problems, the most important and challenging of which is to prevent the inhibition of the cytotoxic effects of NK cells. NK cells are functionally inhibited in the GBM tumor microenvironment. Kozlowska et al. (15) show that GBM stem cells are highly susceptible to NK-mediated killing, but after differentiation of these stem cells, anergic NK cells fail to control GBM tumor growth because of the release of IL-6 and IL-8.

CAR-NK therapy is one of the latest developments in treating GBM. One group of investigators found that ErbB2 protein expression was elevated in a large proportion of GBM samples and used ErbB2/HER2-specific NK cells to target GBM (16), proposing sustainably expanded “CAR-NK” cells—human NK cells that express ErbB2-specific chimeric antigen receptors. The *in vitro* and *in vivo* effects of these CAR-NK cells on GBM cell culture and orthotopic GBM xenograft models as well as the therapeutic effects of NK-92/5.28 cells on endogenous antitumor immunity were also confirmed. Murakami et al. (17) also propose a method comprising a novel NK cell line carrying a chimeric immune antigen receptor (CAR-KHYG-1) to target epidermal growth factor receptor variant III (EGFRvIII) and induce antitumor effects in GBM cells. Han et al. (18) reveal that CAR-redirected NK cells effectively target wt EGFR and EGFRvIII to treat GBM and demonstrate that intracranial application of NK-92-EGFR-CAR cells can effectively inhibit tumor growth, which is a prospective clinical strategy for the treatment of GBM.

In conclusion, owing to the large number of studies on targeted NK cell therapy for GBM in progress, it seems that this treatment modality has a good chance of becoming a full-fledged immunotherapy regimen. However, most of these studies have not made any profound breakthroughs, and the safety and efficacy of adoptive immunotherapy with CAR-NK cells need to be further assessed in clinical trials. Thus, treating GBM with NK cells still has a long way to go.

### Dendritic Cells

DC vaccines have been administered clinically for the treatment of GBM, but the results remain unsatisfactory. Pellegatta et al. (19) propose that DC immunotherapy for GBM might be associated with NK cells, that DC vaccines induced significant and sustained NK cell activation, and that the increase in their response had a significant correlation with prolonged patient survival. Dusoswa et al. (20) selected Siglec-9 ligands highly expressed on GBM extracellular vesicles and modified these vesicles with a receptor to promote Siglec binding on the vesicle surface, thereby achieving efficient targeting of adjuvant DCs to GBM and enhancing their potential as anticancer vaccines.

Vaccines are based on DCs containing peptides that represent one or more specific tumor antigens or whole lysates as a source of multiple antigens. However, factors such as the immunosuppressive microenvironment, lack of appropriate specific epitopes, and cancer immunoediting may limit their efficacy (21).

The activation of DCs can be driven by GBM stem cells and a mixture of monocytes, such as CD34-, CD45-, and CD56-positive cells from allogeneic umbilical cord blood (UCB) (22). Eiraku et al. (23) study the interaction of DCs with CD8+ T cells as well as with Vγ9γδ T cells and Vα24NKT cells. Immunocyte therapy based on DCs interacting with GBM lysate (24) is also a promising treatment for GBM.

A large body of literature has indicated that, because DC vaccines themselves are less toxic and do not have many adverse effects, they may become a new hope for the treatment of GBM.

### Tumor-Associated Macrophages

Only a few studies have shown that macrophages directly play an immune-related role in the treatment of GBM, and Sun et al. (25) find that inhibiting Romo1 in combination with anti-PD-1 immunotherapy significantly improved the prognosis of GBM patients and particularly enhanced the function of macrophages.

Hallmark indicators of genetic alterations in GBM are amplification of EGFR and EGFRvIII, and investigators have proposed a pathway in which EGFR in combination with EGFRvIII induces macrophage infiltration by upregulating the expression of the chemokine CCL2 (26).

Most existing studies on TAMs have focused on the secretion of cytokines in the GBM microenvironment, promoting GBM progression. Herting et al. (27) find in their study that coculturing TAMs derived from bone marrow with primary GBM cells promoted the upregulation of the cytokine IL-1, which is detrimental to the tumor-killing effect of NK cells and T cells.

TAMs promote the growth of GBM by secreting pleiotropic phosphorus and promoting PTPRZ1 signaling in GBM stem cells (28). In addition, a similar study indicated that Wnt-induced signaling protein 1 (WISP1) secreted from GBM stem cells promotes the survival of both GBM stem cells and TAMs (phagocytes) to establish a tumorigenic microenvironment (29).

In addition, research on macrophages has not been consistent. Here, we simply provide examples of the following recent studies. Macrophage-associated cytokines are used as prognostic indicators of GBM, and increased IL-6 levels predict poor prognosis (30); Wei et al. (31) find that osteopontin (OPN) is an important chemokine for recruiting macrophages into GBM. Cui et al. (32) reported the importance of macrophage-associated immunosuppression in GBM angiogenesis. Although these discoveries have not been fully elaborated upon, they provide new ideas for the treatment of GBM with macrophages, indicating that macrophages play multiple roles and are expected to be applied in other aspects.

In fact, there are both advantages and disadvantages of the use of macrophages in immunotherapy. We found that many studies have proven that macrophages have an adverse effect on the prognosis of GBM. Therefore, we believe that how to apply macrophages in the future to maintain their advantages and avoiding their disadvantages will become a new research focus.

### Mast Cells

Põlajeva et al. (33) propose that the accumulation of mast cells (MCs) in GBM tumors might be related to the levels of stem cell factor and the chemokine CXCL12; Attarha et al. (34) demonstrate that MCs respond to multiple signals in a glioma grade-dependent manner to infiltrate mouse and human gliomas and induce the differentiation of glioma cells. Roy et al. (35) use the degree of recruitment of MCs as a potential biomarker for grading GBM.

## Application of Immune Cell Therapy for GBM

The GBM immunotherapy category includes adoptive T cell immunotherapy, CAR-T immunotherapy, DC tumor vaccines, immune checkpoint blockade, monoclonal antibodies, and cytokine therapy.

### Adoptive T Cell Immunotherapy

Adoptive lymphocyte transfer (ALT) is an antigen-specific treatment during which either tumor-infiltrating lymphocytes (TILs) are obtained from tumor specimens or T cells are isolated from peripheral blood mononuclear cells (PBMCs), expanded *in vitro* against tumor antigens, and systemically applied or directly injected into the tumor site (36). Schuessler et al. (37) report the successful expansion of cytomegalovirus-specific T cells from 13 of 19 patients with recurrent GBM; moreover, 4 of the 10 patients who completed the treatment remained tumor-free during the study period.

Currently, multiple clinical trials have used ALT therapy in GBM patients (NCT01082926, NCT00331526, NCT01588769, NCT00003185, and NCT00730613), and these studies have confirmed the safety and feasibility of ALT therapy (38).

More recently, there has been progress in a clinical trial involving adoptive cellular immunotherapies (ACT), which has shown that CMV-specific ACT can effectively delay or even prevent the recurrence of GBM, which indicates that a favorable T cell gene signature is associated with the improvement in therapeutic efficacy and prolonged survival (39).

### CAR-T Immunotherapy

CAR-T immunotherapy is a precisely targeted therapy for the treatment of tumors, which transduces a CAR into T cells to create CAR-T cells (40), after which they are expanded to large numbers *in vitro* and then reinjected into the patient, prompting B cells to produce antibodies and specifically recognize antigens, which, in turn, kill the tumors (41).

CAR-T immunotherapy has the capacity to cross the BBB and can safely and effectively reach tumor cells that cannot be accessed surgically (42). Brown et al. (38) treated a patient with recurrent GBM by using CAR-T cells and found that IL13R-2 was a useful immunotherapeutic target in GBM. Although this therapy has been recognized by many patients, and 4 categories have been clinically approved in China, it is still not considered a conventional treatment.

After reviewing many studies on the subject, we deduced that CAR-T therapy has not made a breakthrough in the treatment of solid tumors in recent years, and there are many unsolved issues, especially in terms of CAR-T cells entering the microenvironment of solid tumors, such as GBM, maintaining their viability and ability to rapidly and accurately identify tumor cells, and overcoming immunosuppression. Therefore, if the issue of tumor microenvironment inhibition of the CAR-T cell therapeutic effects can be solved at this stage, it will have a great impact on the immunotherapy of solid tumors.

### Dendritic Cell Vaccines

The production of DC vaccines includes isolating DCs from patients, loading the cells with tumor antigens, culturing the DCs with cytokines to induce maturation, and reinjecting the cells back into the body (43). At present, vaccines are broadly divided into three categories according to different antigens: tumor-associated antigens (TAAs), tumor-specific antigens (TSAs), and tumor lysates (44).

1. *TAAs are ubiquitous but are expressed at higher levels in tumor cells than in healthy cells, so TAA vaccines are easy to develop and have good targeting*. At present, the clinical application of TAA-based DC vaccines is limited, mainly due to the following reasons: 1. There are few known TAAs, 2. the consistency of TAA expression marks its own limitations, and 3. TAA vaccines may not induce the best immune response due to immune tolerance (44). Wen et al. (45) discovered that ICT-107 vaccination in patients with newly diagnosed GBM developed good tolerance and significantly improved survival by 2.2 months.
2. *TSAs are unique to tumor cells and, unlike TAAs in tumor cells and normal cells, are usually proteins encoded by mutated genes in tumors*. They are relatively fixed in different types of cancer and patients and can be used as targets for immunotherapy (43). TSA-based DC vaccines may generate an intense targeted inflammatory response against tumor cells while avoiding potential autoimmune responses in other tissues (44). Rindopepimut (46) (Celldex Therapeutics, Hampton, New Jersey, USA), a TSA vaccine, has shown clinical benefits and significant efficacy in phase II clinical trials. However, the phase III clinical trial was terminated early because it was thought that the patients in the study might not reach their primary endpoint.
3. *Vaccination in combination with tumor lysates is usually delivered by autologous lysate-pulsed DCs, which are usually collected from the patient a few days before surgical resection, incubated with the resected tumor lysate, and then reintroduced back into patients via postoperative multiple vaccinations in combination with standardized radiotherapy administration to target residual tumor cells* (44). The phase III trial by Liau et al. (47) demonstrated that the addition of DCVax-L to the standard of care for GBM patients is feasible and safe and prolongs survival. The combination of tumor antigens and a-GalCer in anticancer vaccines can efficiently induce long-lasting immunity by activating iNK T cells (48).

Therefore, the latest research direction of DC vaccines at this stage focuses on combining tumor lysates with DCs, and the mechanism of tumor lysate vaccines on GBM is very likely to be the combined action of multiple immune cells.

## Prospect of Immunotherapy for GBM

### Different Combination Regimens

Immunotherapy for GBM has not been used in a wide range of direct treatments due to its immaturity and our incomplete understanding of the mechanisms, so the main treatment modalities after diagnosis confirmation are surgical resection, radiotherapy, chemotherapy, and various comprehensive treatments. Conventional treatment is maximal gross resection of the tumor followed by radiotherapy and chemotherapy, and maintenance therapy with temozolomide (TMZ) is started 4 weeks after completion of the chemoradiotherapy cycle. This treatment regimen is known the standard of care (SOC). The common chemotherapeutic agent axitinib has been shown by Stephanie Du Four et al. to have a favorable effect on immune function (49).

There are currently several main GBM vaccines available: 1. autologous monocyte vaccine, 2. peptide-based tumor vaccine, 3. nucleotide-based tumor vaccine, and 4. cell line–based tumor vaccine. Each of them requires coculture of the corresponding cells with surgically resected tumor cells under different conditions to achieve immunization against tumor cells. Therefore, it is also necessary to obtain a sufficient number of tumor cells at the time of surgery.

CRS-T is a genetically modified chimera-switched receptor T cell therapy targeting PD-1. After intravenous infusion of CSR-T cells, the levels of IFN-γ and IL-6 in peripheral blood increase with the number of reinfused cells, and local intracranial injection of CSR-T cells is often more effective than intravenous injection (50).

Immunotherapy with DC vaccines has been associated with adverse effects of immunotherapy in newly diagnosed patients, and increases in tumor-specific immune responses after vaccination, including immune cell proliferation and cytokine production, can be detected (51). For patients with recurrent GBM, elevated levels of chemoresistance-associated peptides (CAPs) and/or cytoplasmic accumulation can be observed in fusion cells generated after cervical implantation of autologous glioma cells and DCs, and a specific immune response to CAPs can also be observed, which promotes an antitumor response in patients (52).

Cytokine-induced killer (CIK) cells are nonhistocompatibility (MHC)-restricted lymphotoxic cells that can be produced from PBMCs under the induction of interferon (IFN)-γ, IL-2, and CD3 monoclonal antibodies (CD3 mAb) and have a high proliferation rate and antitumor activity. Chemoradiation with CIK cells plus the standard radiotherapy-TMZ regimen shows no significant difference in survival but improves progression-free survival compared with that of TMZ alone; unfortunately, no evidence of improved overall survival was found (53).

Rindopepimut (also known as CDX-110) is a peptide-based vaccine against EGFRvIII, an EGFR variate with a deletion mutation, and the addition of rindopepimut to a standardized course did not increase survival in patients with newly diagnosed GBM in clinical trials. Further exploration of the effect of immunotherapy in future treatment combinations containing rindopepimut may be required (54).

Different vaccines are injected in different ways, and some cellular vaccines can be injected directly into the surgical cavity. After surgical resection of GBM, patients undergoing the SOC followed by local injection of an immune-stimulating oligonucleotide containing an unmethylated cytosine-guanosine motif (CpG-ODN) immediately around the surgical cavity were more likely to develop fever and postoperative hematoma after surgery than patients who received the SOC alone with similar incidences of other adverse events. Overall, this vaccine did not improve survival in patients with newly diagnosed GBM (55).

In addition, vaccines made from oncolytic viruses are also under investigation. The oncolytic virus aglatimagene besadenovec (AdV-tk) combined with valacyclovir constitutes gene-mediated cytotoxic immunotherapy (GMCI). When combined with the SOC, GMCI may stimulate immune responses in participants with GTR and subtotal resection. However, if the residual tumor burden is too large, the tumor-mediated immunosuppressive effect may mask the effect of GMCI (56).

At present, most immunotherapies can only be performed after patients undergo surgical resection, which is a limitation of immunotherapy. However, as a brain tumor, GBM is very likely to grow in sites that are not suitable for surgical resection and are more likely to recur, and treatment with immune cells or vaccines can achieve the destruction of tumors at sites that are not easily accessible *via* surgery; therefore, if the field of immunotherapy can mature, this treatment modality may replace surgical resection.

### Role of Exosomes in Tumor Immunology

Exosomal vesicles naturally released by tumor cells transmit some molecules to target cells and act as an intercellular signaling pathway between the donor cytoplasm and the lumen of target cells. For example, NK cells release exosomes that express typical NK markers (CD56, etc.), killer proteins, and stimulate antitumor activity (57, 58). Exosomes carrying different RNAs and proteins can be measured in patients as biomarkers to assess disease onset and progression (59).

Barile et al. (60) demonstrate that GBM stem cells could secrete exosomes carrying active vascular endothelial growth factor A (VEGF-A), which could protect VEGF-A from cytokines, proteases, etc., in the tumor microenvironment and maintain the vascular niche. Non-GBM-stem-cell-derived VEGF-A changes with tumor size and treatment, whereas GBM-stem-cell-derived exosomes may be continuously produced and released and cross the BBB.

Skog et al. (61) and Brennan et al. (62) report that EGFRvIII could be detected in the serum exosomes of GBM patients with high diagnostic sensitivity, but EGFRvIII was present in only approximately 25% of GBM patients. Kai et al. (63) demonstrate that PTRF was also present on the exosomal membrane and that PTRF overexpression increased exosome secretion, resulting in increased rates of tumor formation and receptor proliferation *in vitro* and *in vivo*.

A study by Liu et al. (48) shows that, in a DC vaccine, the codelivery of tumor-derived exosomes with α-galactosylceramide (α-GalCer) could effectively improve the tumor microenvironment by balancing the release of immunosuppressive factors and immunostimulatory factors. Zhu et al. (64) find that exosomes secreted by NK cells could specifically localize to GBM tumors and that NK-Exos contain FasL, perforin, granzyme B, and TNF-α, thereby inducing proapoptotic signals and triggering cell death in tumors. Chen et al. (65) propose that exosomes could interact with target cells with barrier cells. It is a new promising therapeutic agent for the treatment of refractory GBM.

Exosomal transfer of miRNAs or miRNA inhibitors to tumor cells has emerged as a new approach to deliver miRNAs that can target cancer. MiRNA-21 is overexpressed in GBM, which can improve the proliferation and malignant metastatic behavior of tumor cells (66, 67). Sponge constructs are designed to bind to their complementary miRNAs (one or more) or their seed sequences, thereby preventing miRNA binding to their biological targets.

GBM cells use exosomes to communicate with the tumor microenvironment and promote their proliferation, invasion, and metastasis (68). One of the major challenges of exosome-based therapeutic approaches is the low productivity of exosomes. Therefore, effective methods that can produce exosomes on a large scale are needed. Watson et al. (69) propose that the use of hollow fiber bioreactors could increase the yield of exosomes by 5- to 10-fold.

Although exosomes have been a research hot spot in recent years, the still-limited data on the use of exosomes as a targeted therapy for GBM is far from mature. There is no denying the fact that using GBM-derived and -targeted exosomes is a very good idea, but more research and clinical trials are needed to truly determine their effect in patients, which can take years. Thus, it is difficult to judge whether this approach is worth pursuing, but at this stage, we are optimistic that ongoing clinical trials will provide a preliminary basis for the use of exosomes in immune therapy.

### Research Focuses and Prospects of Other Immune Cell Therapies

One popular direction is the implementation of combined antigen immunotherapies with the latest focus on CAR-Ts and “bispecific T cell engagers” (BiTEs) (70, 71), that is, using antibodies such as BiTEs to bind both T cells and tumor cells and create a molecular bridge that induces T cells to kill GBM cells.

In addition, following CAR-T therapy, a series of CAR immunotherapies have been investigated, such as CAR-NK cells (72) and CAR-macrophages. In the field of cancer immunotherapy, CAR-T cell therapy and PD-1 inhibitors are the most high-profile members, but in addition to T cells, the functions of NK cells and macrophages should not be ignored.

## Summary

In summary, it is of great significance to explore the mechanisms of action of immunotherapy in depth for the treatment of GBM and to specifically study these mechanisms of action in each type of immune cell. In addition, immunotherapy has incomparable advantages to surgical treatment, radiotherapy, and chemotherapy.

We believe that the most valuable applications of immunotherapy for the treatment of GBM are CAR-T cell therapy and DC vaccines. These two methods have a large body of preliminary data and have undergone clinical trials and applications. Although there may be some issues, any problems that arise are expected to be resolved in follow-up studies. Therefore, in the process of GBM occurrence and expansion, the use of appropriate treatment options and attempts to use immunotherapy are conducive to not only the broad killing of cancer cells but also mediating the occurrence and expansion rate of GBM. This activity may strongly contribute to the treatment of GBM and even other types of cancer.

## Author Contributions

XK, HT, and WG contributed to the conception and design of the study. XK, YZ, WG, XC, and WH wrote the first draft of the manuscript. HL, BH, and ZH wrote sections of the manuscript. All authors contributed to the article and approved the submitted version.

## Funding

This study was funded by the National Natural Science Foundation of China (81774109, 81973620), the Natural Science Foundation of Zhejiang Province (Y19H310028), the Wenzhou Science and Technology Project (ZY2019015), and the Zhejiang Public Welfare Technology Research Plan (LGD20H290002).

## Conflict of Interest

The authors declare that the research was conducted in the absence of any commercial or financial relationships that could be construed as a potential conflict of interest.

## References

[1] Alexander BM Cloughesy TF Adult Glioblastoma. J Clin Oncol (2017) 35(21):2402–9. 10.1200/JCO.2017.73.0119 28640706

[2] Chaichana KL Parker SL Olivi A Quiñones-Hinojosa A Long-term seizure outcomes in adult patients undergoing primary resection of malignant brain astrocytomas. Clinical article. J Neurosurg (2009) 111(2):282–92. 10.3171/2009.2.JNS081132 19344222

[3] Lim M Xia YX Bettegowda C Weller M Current state of immunotherapy for glioblastoma. Nat Rev Clin Oncol (2018) 15(7):422–42. 10.1038/s41571-018-0003-5 29643471

[4] Jackson CM Choi J Lim M Mechanisms of immunotherapy resistance: lessons from glioblastoma. Nat Immunol (2019) 20(9):1100–9. 10.1038/s41590-019-0433-y 31358997

[5] Brown NF Carter TJ Ottaviani D Mulholland P Harnessing the immune system in glioblastoma. Br J Cancer (2018) 119(10):1171–81. 10.1038/s41416-018-0258-8 PMC6251037 30393372

[6] Chang LS Barroso-Sousa R Tolaney SM Hodi FS Kaiser UB Min L Endocrine Toxicity of Cancer Immunotherapy Targeting Immune Checkpoints. Endocr Rev (2019) 40(1):17–65. 10.1210/er.2018-00006 30184160 PMC6270990

[7] Luoto S Hermelo I Vuorinen EM Hannus P Kesseli J Nykter M Computational Characterization of Suppressive Immune Microenvironments in Glioblastoma. Cancer Res (2018) 78(19):5574–85. 10.1158/0008-5472.CAN-17-3714 29921698

[8] Golán I Rodríguez de la FL Costoya JA NK Cell-Based Glioblastoma Immunotherapy. Cancers (Basel) (2018) 10(12):1–32. 10.3390/cancers10120522 PMC6315402 30567306

[9] Burger MC Zhang CC Harter PN Romanski A Strassheimer F Senft C CAR-Engineered NK Cells for the Treatment of Glioblastoma: Turning Innate Effectors Into Precision Tools for Cancer Immunotherapy. Front Immunol (2019) 10:2683. 10.3389/fimmu.2019.02683 31798595 PMC6868035

[10] Gras NA Kmiecik J Leiss L Zelkowski M Engelsen A Bruserud Ø. NK cells with KIR2DS2 immunogenotype have a functional activation advantage to efficiently kill glioblastoma and prolong animal survival. J Immunol (2014) 193(12):6192–206. 10.4049/jimmunol.1400859 PMC4259203 25381437

[11] Lee SJ Kang WY Yoon Y Jin JY Song HJ Her JH Natural killer (NK) cells inhibit systemic metastasis of glioblastoma cells and have therapeutic effects against glioblastomas in the brain. BMC Cancer (2015) 15:1–28. 10.1186/s12885-015-2034-y 26704632 PMC4690248

[12] Dominguez-Valentin M Gras NA Rahman AM Kumar S Retière C Ulvestad E Identification of a Natural Killer Cell Receptor Allele That Prolongs Survival of Cytomegalovirus-Positive Glioblastoma Patients. Cancer Res (2016) 76(18):5326–36. 10.1158/0008-5472.CAN-16-1162 27406829

[13] Yvon ES Burga R Powell A Cruz CR Fernandes R Barese C Cord blood natural killer cells expressing a dominant negative TGF-β receptor: Implications for adoptive immunotherapy for glioblastoma. Cytotherapy (2017) 19(3):408–18. 10.1016/j.jcyt.2016.12.005 28109751

[14] Shevtsov M Pitkin E Ischenko A Stangl S Khachatryan W Galibin O Ex vivo Hsp70-Activated NK Cells in Combination With PD-1 Inhibition Significantly Increase Overall Survival in Preclinical Models of Glioblastoma and Lung Cancer. Front Immunol (2019) 10:454. 10.3389/fimmu.2019.00454 30967859 PMC6439337

[15] Kozlowska AK Tseng H-C Kaur K Topchyan P Inagaki A Bui VT Resistance to cytotoxicity and sustained release of interleukin-6 and interleukin-8 in the presence of decreased interferon-γ after differentiation of glioblastoma by human natural killer cells. Cancer Immunol Immunother (2016) 65(9):1085–97. 10.1007/s00262-016-1866-x PMC4996719 27439500

[16] Zhang CC Burger MC Jennewein L Genßler S Schönfeld K Zeiner P ErbB2/HER2-Specific NK Cells for Targeted Therapy of Glioblastoma. J Natl Cancer Inst (2016) 108(5):1–12. 10.1093/jnci/djv375 26640245

[17] Murakami T Nakazawa T Natsume A Nishimura F Nakamura M Matsuda R Novel Human NK Cell Line Carrying CAR Targeting EGFRvIII Induces Antitumor Effects in Glioblastoma Cells. Anticancer Res (2018) 38(9):5049–56. 10.21873/anticanres.12824 30194149

[18] Han JF Chu JH Keung CW Zhang JY Wang YW Cohen JB CAR-Engineered NK Cells Targeting Wild-Type EGFR and EGFRvIII Enhance Killing of Glioblastoma and Patient-Derived Glioblastoma Stem Cells. Sci Rep (2015) 5:11483. 10.1038/srep11483 26155832 PMC4496728

[19] Pellegatta S Eoli M Cuccarini V Anghileri E Pollo B Pessina S Survival gain in glioblastoma patients treated with dendritic cell immunotherapy is associated with increased NK but not CD8 T cell activation in the presence of adjuvant temozolomide. Oncoimmunology (2018) 7(4):e1412901. 10.1080/2162402X.2017.1412901 29632727 PMC5889286

[20] Dusoswa SA Horrevorts SK Ambrosini M Kalay H Paauw NJ Nieuwland R Glycan modification of glioblastoma-derived extracellular vesicles enhances receptor-mediated targeting of dendritic cells. J Extracell Vesicles (2019) 8(1):1648995. 10.1080/20013078.2019.1648995 31489145 PMC6713149

[21] Finocchiaro G Pellegatta S Immunotherapy with dendritic cells loaded with glioblastoma stem cells: from preclinical to clinical studies. Cancer Immunol. Immunother (2016) 65(1):101–9. 10.1007/s00262-015-1754-9 PMC11029491 26377689

[22] Yurtsever A Haydaroglu A Biray AC Gunduz C Oktar N Dalbasti T Assessment of genetic markers and glioblastoma stem-like cells in activation of dendritic cells. Hum Cell (2013) 26(3):105–13. 10.1007/s13577-013-0065-8 23737374

[23] Eiraku Y Terunuma H Yagi M Deng X Nicol AJ Nieda M Dendritic cells cross-talk with tumour antigen-specific CD8 T cells, Vγ9γδT cells and Vα24NKT cells in patients with glioblastoma multiforme and in healthy donors. Clin Exp Immunol (2018) 194(1):54–66. 10.1111/cei.13185 30009488 PMC6156812

[24] Nava S Lisini D Pogliani S Dossena M Bersano A Pellegatta S Safe and Reproducible Preparation of Functional Dendritic Cells for Immunotherapy in Glioblastoma Patients. Stem Cells Transl Med (2015) 4(10):1164–72. 10.5966/sctm.2015-0091 PMC4572908 26273063

[25] Sun G Cao Y Qian C Wan Z Zhu J Guo J Romo1 is involved in the immune response of glioblastoma by regulating the function of macrophages. Aging (Albany NY) (2020) 12:1114–27. 10.18632/aging.102648 PMC7053633 31945745

[26] An Z Knobbe-Thomsen CB Wan X Fan QW Reifenberger G Weiss WA Correction: EGFR Cooperates with EGFRvIII to Recruit Macrophages in Glioblastoma. Cancer Res (2019) 79(21):5681. 10.1158/0008-5472.CAN-19-2800 31676678

[27] Herting CJ Chen Z Maximov V Duffy A Szulzewsky F Shayakhmetov DM Tumour-associated macrophage-derived interleukin-1 mediates glioblastoma-associated cerebral oedema. Brain (2019) 142(12):3834–51. 10.1093/brain/awz331 PMC6906596 31665239

[28] Shi Y Ping YF Zhou W He ZC Chen C Bian BSJ Tumour-associated macrophages secrete pleiotrophin to promote PTPRZ1 signalling in glioblastoma stem cells for tumour growth. Nat Commun (2017) 8:15080. 10.1038/ncomms15080 28569747 PMC5461490

[29] Tao W Chu C Zhou W Huang Z Zhai K Fang X Dual Role of WISP1 in maintaining glioma stem cells and tumor-supportive macrophages in glioblastoma. Nat Commun (2020) 11(1):3015. 10.1038/s41467-020-16827-z 32541784 PMC7295765

[30] Hori T Sasayama T Tanaka K Koma Y II Nishihara M Tanaka H Tumor-associated macrophage related interleukin-6 in cerebrospinal fluid as a prognostic marker for glioblastoma. J Clin Neurosci (2019) 68:281–9. 10.1016/j.jocn.2019.07.020 31327593

[31] Wei J Marisetty A Schrand B Gabrusiewicz K Hashimoto Y Ott M Osteopontin mediates glioblastoma-associated macrophage infiltration and is a potential therapeutic target. J Clin Invest (2019) 129(1):137–49. 10.1172/JCI121266 PMC6307970 30307407

[32] Cui X Morales RTT Qian W Wang H Gagner JP Dolgalev I Hacking macrophage-associated immunosuppression for regulating glioblastoma angiogenesis. Biomaterials (2018) 161:164–78. 10.1016/j.biomaterials.2018.01.053 PMC8059366 29421553

[33] Põlajeva J Sjösten AM Lager N Kastemar M Waern I Alafuzoff I Mast cell accumulation in glioblastoma with a potential role for stem cell factor and chemokine CXCL12. PLoS One (2011) 6(9):e25222. 10.1371/journal.pone.0025222 21949886 PMC3176317

[34] Attarha S Roy A Westermark B Tchougounova E Mast cells modulate proliferation, migration and stemness of glioma cells through downregulation of GSK3β expression and inhibition of STAT3 activation. Cell Signal (2017) 37:81–92. 10.1016/j.cellsig.2017.06.004 28600192

[35] Roy A Attarha S Weishaupt H Edqvist PH Swartling FJ Bergqvist M Serglycin as a potential biomarker for glioma: association of serglycin expression, extent of mast cell recruitment and glioblastoma progression. Oncotarget (2017) 8(15):24815–27. 10.18632/oncotarget.15820 PMC5421891 28445977

[36] Farber SH Elsamadicy AA Atik AF Suryadevara CM Chongsathidkiet P Fecci PE The Safety of available immunotherapy for the treatment of glioblastoma. Expert Opin Drug Saf (2017) 16(3):277–87. 10.1080/14740338.2017.1273898 PMC5404815 27989218

[37] Schuessler A Smith C Beagley L Boyle GM Rehan S Matthews K Autologous T-cell therapy for cytomegalovirus as a consolidative treatment for recurrent glioblastoma. Cancer Res (2014) 74(13):3466–76. 10.1158/0008-5472.CAN-14-0296 24795429

[38] Brown CE Alizadeh D Starr R Weng L Wagner JR Naranjo A Regression of Glioblastoma after Chimeric Antigen Receptor T-Cell Therapy. N Engl J Med (2016) 375(26):2561–9. 10.1056/NEJMoa1610497 PMC5390684 28029927

[39] Smith C Lineburg KE Martins JP Ambalathingal G Neller MA Morrison B Autologous CMV-specific T cells are a safe adjuvant immunotherapy for primary glioblastoma multiforme. J Clin Invest (2020). 10.1172/JCI138649 PMC7598048 32750039

[40] Srivastava S Riddell SR Engineering CAR-T cells: Design concepts. Trends Immunol (2015) 36(8):494–502. 10.1016/j.it.2015.06.004 26169254 PMC4746114

[41] Bagley SJ Desai AS Linette GP June CH O’Rourke DM CAR T-cell therapy for glioblastoma: recent clinical advances and future challenges. Neuro-oncology (2018) 20(11):1429–38. 10.1093/neuonc/noy032 PMC6176794 29509936

[42] Filley AC Henriquez M Dey M CART Immunotherapy: Development, Success, and Translation to Malignant Gliomas and Other Solid Tumors. Front Oncol (2018) 8:453. 10.3389/fonc.2018.00453 30386740 PMC6199385

[43] Kong Z Wang Y Ma W Vaccination in the immunotherapy of glioblastoma. Hum Vaccin Immunother (2018) 14(2):255–68. 10.1080/21645515.2017.1388481 PMC5806656 29087782

[44] Wilcox JA Ramakrishna R Magge R Immunotherapy in Glioblastoma. World Neurosurg (2018) 116:518–28. 10.1016/j.wneu.2018.04.020 30049046

[45] Wen PY Reardon DA Armstrong TS Phuphanich S Aiken RD Landolfi JC A Randomized Double-Blind Placebo-Controlled Phase II Trial of Dendritic Cell Vaccine ICT-107 in Newly Diagnosed Patients with Glioblastoma. Clin Cancer Res (2019) 25(19):5799–807. 10.1158/1078-0432.CCR-19-0261 PMC8132111 31320597

[46] Elsamadicy AA Chongsathidkiet P Desai R Woroniecka K Farber SH Fecci PE Prospect of rindopepimut in the treatment of glioblastoma. Expert Opin Biol Ther (2017) 17(4):507–13. 10.1080/14712598.2017.1299705 PMC5787389 28274144

[47] Liau LM Ashkan K Tran DD Campian JL Trusheim JE Cobbs CS First results on survival from a large Phase 3 clinical trial of an autologous dendritic cell vaccine in newly diagnosed glioblastoma. J Transl Med (2018) 16(1):142. 10.1186/s12967-018-1507-6 29843811 PMC5975654

[48] Liu H Chen L Liu J Meng HX Zhang R Ma L Co-delivery of tumor-derived exosomes with alpha-galactosylceramide on dendritic cell-based immunotherapy for glioblastoma. Cancer Lett (2017) 411:182–90. 10.1016/j.canlet.2017.09.022 28947140

[49] Du FS Maenhout SK Benteyn D De KB Duerinck J Thielemans K Disease progression in recurrent glioblastoma patients treated with the VEGFR inhibitor axitinib is associated with increased regulatory T cell numbers and T cell exhaustion. Cancer Immunol Immunother (2016) 65(6):727–40. 10.1007/s00262-016-1836-3 PMC11029796 27098427

[50] Guo JX Wu CX Wang PF Li ZJ Han S Jin W Bioactivity and safety of chimeric switch receptor T cells in glioblastoma patients. Front Biosci (Landmark Ed) (2019) 24:1158–66. 10.2741/4772 31136972

[51] Inogés S Tejada S Lopez-Diaz de Cerio Ascensión Gállego PLJ Espinós J Idoate MA A phase II trial of autologous dendritic cell vaccination and radiochemotherapy following fluorescence-guided surgery in newly diagnosed glioblastoma patients. J Transl Med (2017) 15(1):104. 10.1186/s12967-017-1202-z 28499389 PMC5427614

[52] Akasaki Y Kikuchi T Homma S Koido S Ohkusa T Tasaki T Phase I/II trial of combination of temozolomide chemotherapy and immunotherapy with fusions of dendritic and glioma cells in patients with glioblastoma. Cancer Immunol Immunother (2016) 65(12):1499–509. 10.1007/s00262-016-1905-7 PMC11028634 27688162

[53] Kong DS Nam DH Kang SH Lee JW Chang JH Kim JH Phase III randomized trial of autologous cytokine-induced killer cell immunotherapy for newly diagnosed glioblastoma in Korea. Oncotarget (2017) 8(4):7003–13. 10.18632/oncotarget.12273 PMC5351686 27690294

[54] Weller M Butowski N Tran DD Recht LD Lim M Hirte H Rindopepimut with temozolomide for patients with newly diagnosed, EGFRvIII-expressing glioblastoma (ACT IV): a randomised, double-blind, international phase 3 trial. Lancet Oncol (2017) 18(10):1373–85. 10.1016/S1470-2045(17)30517-X 28844499

[55] Ursu R Carpentier A Metellus P Lubrano V Laigle-Donadey F Capelle L Intracerebral injection of CpG oligonucleotide for patients with de novo glioblastoma-A phase II multicentric, randomised study. Eur J Cancer (2017) 73:30–7. 10.1016/j.ejca.2016.12.003 28142059

[56] Wheeler LA Manzanera AG Bell SD Cavaliere R McGregor JM Grecula JC Phase II multicenter study of gene-mediated cytotoxic immunotherapy as adjuvant to surgical resection for newly diagnosed malignant glioma. Neuro-oncology (2016) 18(8):1137–45. 10.1093/neuonc/now002 PMC4933478 26843484

[57] Parolini I Federici C Raggi C Lugini L Palleschi S De MA Microenvironmental pH is a key factor for exosome traffic in tumor cells. J Biol Chem (2009) 284(49):34211–22. 10.1074/jbc.M109.041152 PMC2797191 19801663

[58] Shoae-Hassani A Hamidieh AA Behfar M Mohseni R Mortazavi-Tabatabaei SA Asgharzadeh S NK Cell-derived Exosomes From NK Cells Previously Exposed to Neuroblastoma Cells Augment the Antitumor Activity of Cytokine-activated NK Cells. J Immunother (2017) 40(7):265–76. 10.1097/CJI.0000000000000179 PMC7543683 28622272

[59] Treps L Perret R Edmond S Ricard D Gavard J Glioblastoma stem-like cells secrete the pro-angiogenic VEGF-A factor in extracellular vesicles. J Extracell Vesicles (2017) 6(1):1359479. 10.1080/20013078.2017.1359479 28815003 PMC5549846

[60] Barile L Vassalli G Exosomes: Therapy delivery tools and biomarkers of diseases. Pharmacol Ther (2017) 174:63–78. 10.1016/j.pharmthera.2017.02.020 28202367

[61] Skog J Würdinger T van RS Meijer DH Gainche L Sena-Esteves M Glioblastoma microvesicles transport RNA and proteins that promote tumour growth and provide diagnostic biomarkers. Nat Cell Biol (2008) 10(12):1470–6. 10.1038/ncb1800 PMC3423894 19011622

[62] Brennan CW Verhaak RGW McKenna A Campos B Noushmehr H Salama SR The somatic genomic landscape of glioblastoma. Cell (2013) 155(2):462–77. 10.1016/j.cell.2013.09.034 PMC3910500 24120142

[63] Huang K Fang C Yi K Liu X Qi H Tan Y The role of PTRF/Cavin1 as a biomarker in both glioma and serum exosomes. Theranostics (2018) 8(6):1540–57. 10.7150/thno.22952 PMC5858166 29556340

[64] Zhu L Kalimuthu S Gangadaran P Oh JM Lee HW Baek SH Exosomes Derived From Natural Killer Cells Exert Therapeutic Effect in Melanoma. Theranostics (2017) 7(10):2732–45. 10.7150/thno.18752 PMC5558565 28819459

[65] Chen CC Liu L Ma F Wong CW Guo XE Chacko JV Elucidation of Exosome Migration across the Blood-Brain Barrier Model In Vitro. Cell Mol Bioeng (2016) 9(4):509–29. 10.1007/s12195-016-0458-3 PMC5382965 28392840

[66] Monfared H Jahangard Y Nikkhah M Mirnajafi-Zadeh J Mowla SJ Potential Therapeutic Effects of Exosomes Packed With a miR-21-Sponge Construct in a Rat Model of Glioblastoma. Front Oncol (2019) 9:782. 10.3389/fonc.2019.00782 31482067 PMC6710330

[67] Chai C Song LJ Han SY Li XQ Li M MicroRNA-21 promotes glioma cell proliferation and inhibits senescence and apoptosis by targeting SPRY1 via the PTEN/PI3K/AKT signaling pathway. CNS Neurosci Ther (2018) 24(5):369–80. 10.1111/cns.12785 PMC6489721 29316313

[68] Kucharzewska P Christianson HC Welch JE Svensson KJ Fredlund E Ringnér M Exosomes reflect the hypoxic status of glioma cells and mediate hypoxia-dependent activation of vascular cells during tumor development. Proc Natl Acad Sci U S A (2013) 110(18):7312–7. 10.1073/pnas.1220998110 PMC3645587 23589885

[69] Watson DC Bayik D Srivatsan A Bergamaschi C Valentin A Niu G Efficient production and enhanced tumor delivery of engineered extracellular vesicles. Biomaterials (2016) 105:195–205. 10.1016/j.biomaterials.2016.07.003 27522254 PMC7156278

[70] Choi BD Yu X Castano AP Bouffard AA Schmidts A Larson RC CAR-T cells secreting BiTEs circumvent antigen escape without detectable toxicity. Nat Biotechnol (2019) 37(9):1049–58. 10.1038/s41587-019-0192-1 31332324

[71] Abbott RC Cross RS Jenkins MR Finding the Keys to the CAR: Identifying Novel Target Antigens for T Cell Redirection Immunotherapies. Int J Mol Sci (2020) 21(2):1–16. 10.3390/ijms21020515 PMC7014258 31947597

[72] Li Ye Hermanson DL Moriarity BS Kaufman DS Human iPSC-Derived Natural Killer Cells Engineered with Chimeric Antigen Receptors Enhance Anti-tumor Activity. Cell Stem Cell (2018) 23(2):181–92.e5. 10.1016/j.stem.2018.06.002 30082067 PMC6084450

